# Associations of serious physical injuries with posttraumatic stress and depressive symptoms: a cross-sectional survey among university students in 26 countries

**DOI:** 10.1186/s40359-020-00501-6

**Published:** 2020-12-09

**Authors:** Supa Pengpid, Karl Peltzer

**Affiliations:** 1 ASEAN Institute for Health Development, Mahidol University, Salaya, Phutthamonthon, Nakhon Pathom, Thailand; 2 Department of Research Administration and Development, University of Limpopo, Turfloop, Mankweng, South Africa; 3 Department of Psychology, University of the Free State, Bloemfontein, South Africa

**Keywords:** Injuries, Stress disorders, Depressive symptoms, Students, Americas, Africa, Asia

## Abstract

**Background:**

Evidence of the relationship between serious physical injury and poor mental health among university students from low- and middle-income countries is limited. The aim of the study is to assess the association between serious physical injury and posttraumatic stress disorder (PTSD) and depressive symptoms in university students from low- and middle-income countries.

**Methods:**

In a cross-sectional survey, 18,382 university students from 26 countries responded to a short screening scale for DSM-IV PTSD, Center for Epidemiologic Studies Depression Scale as well as questions on injury and sociodemographics.

**Results:**

The overall prevalence of past 12-month serious physical injury was 24.7%. In adjusted logistic regression analysis, compared to having no past 12-month serious physical injury, having a past 12-month serious injury was associated with 1.35 (95% CI 1.18, 1.56) times higher odds for PTSD symptoms and 1.49 (95% CI 1.32, 1.67) times higher odds for depressive symptoms in university students.

**Conclusion:**

Compared to students who had not sustained a serious physical injury in the past 12 months, students with an injury had significantly higher PTSD and depressive symptoms. Mental health support of students who sustained physical injuries may prevent PTSD and depressive symptoms.

## Background

Injury, depression and anxiety disorders, including posttraumatic stress disorder (PTSD), are one of the main causes of disability worldwide, in particular in low- and middle-income countries [1, 2]. University students may be particularly vulnerable to injury, PTSD, and depressive symptoms. For example, in a study among university students in 26 countries the prevalence of past 12-month injury was 25.2% [3]. In a review of 37 studies among university students in 20 countries, the overall prevalence of depressive symptoms was 24.4% [4], and in a study among university students from 22 countries, the prevalence of PTSD symptoms was 20.9% [5].

However, there seems to be a lack of research among university students, in particular in low- and middle-income countries, on the association between injury, PTSD and depression symptoms. In systematic reviews based on clinical studies, physical injury was associated with PTSD, anxiety and depression [6, 7]. In an adult population study in Norway, the most common events causing PTSD included, apart from physical and sexual assaults, also injury [8]. In a study among high-school students in Canada, injury was across injury types (violence, transport, and unintentional) was consistently associated with increased depressive symptoms [9], and in a national study among US adolescents, past-year injury increased the odds of past-month anxiety disorders [10]. In a study among adults in 40 low- and middle-income countries, the pooled odds ratio (OR) for depression was 2.04 for other injuries and 1.72 for traffic injury [11]. Based on a study among predominantly older adults in six low- and middle-income countries, the prevalence of past 12-month depression was significantly higher among persons who had any past 12-month injury compared to those without injuries [12], and in an adolescent school survey in 21 low- and middle-income countries, the pooled OR for the association of past 12-month physical injury and past 12-month depressive symptoms was 1.83 [13].

The above studies were conducted among adolescents, adults, and older adults, and most other studies investigating the association between physical injury utilized clinical samples, frequently lacked a control group, often used small sample sizes and were conducted in high-income countries [13], creating the omission of studies among university students in low- and middle-income countries. Previous results may be difficult to extrapolate to university students in low- and middle-income countries, since there may be important differences in the causes of injury and mental health outcomes [13]. For example, compared to those in high-income countries, there may be a greater lack of availability of both care for injuries and mental health in low-resourced countries [13]. Given the high prevalence of physical injury among university students in low resourced settings [3], the analysis of the potential impact of physical injury on mental health outcomes would be of outmost relevance. Therefore, this study is filling gaps in the evidence on the relationship between physical injury, PTSD, and depressive symptoms in a population survey among university students.

Some possible mechanisms at play linking physical injury and PTSD and depressive symptoms may include that the serious physical injury is perceived as a traumatic event leading to PTSD symptoms [6], physical injury may be associated with pain and/or disability increasing the risk for depression [11–13]. This study aimed to identify associations between physical injury, PTSD, and depressive symptoms among university students from 26 predominantly low- and middle-income countries. It was hypothesized that having experienced a physical injury would increase PTSD and depressive symptoms.

## Methods

### Study population and procedures

The study design was a cross-sectional survey of undergraduate students in 26 countries: Bangladesh, Barbados, Cameroon, China, Columbia, Egypt, Grenada, India, Indonesia, Ivory Coast, Jamaica, Kyrgyzstan, Laos, Madagascar, Mauritius, Namibia, Nigeria, Pakistan, Philippines, Russia, Singapore, South Africa, Thailand, Tunisia, Turkey, and Venezuela. The study was initiated through personal or academic contacts of the principal investigators; thus, in each study country one or two universities were purposefully selected. Undergraduate students were sampled by using a quasi-random selection process, which entailed randomly selecting one department from each university faculty, and a random selection was then made from an ordered list of all undergraduate courses offered within the selected department. Trained research assistants then described the study to students within the selected undergraduate class to recruit participants. The inclusion criterion was being present in class at the time of recruitment. The consent form included a written justification of the study and contact details of the local principal investigator were provided to respond to any questions or personal concerns. Trained research assistants administered the paper-based self-administered anonymous questionnaire and made sure that each student had privacy when filling in the questionnaire. The consent forms and questionnaires (without identifying information) were collected separately and placed in different boxes in the front of the room. The questionnaire took about 40 min to complete. It was developed in English, and then translated and back-translated into the languages of the study country following scientific procedures. The study response rate was more than 90% in most countries [14, 15].

### Measures

#### Outcome variables

The outcome variables included PTSD symptoms and depressive symptoms. Past month PTSD symptoms were measured with a 7-item short screening scale for DSM-IV PTSD (Yes, No). The screen was scored by summing the positive responses with scores ranging from 0 to 7 and those who answered affirmatively to at least four of the questions were considered to have a positive screen for PTSD [16], identifying cases of PTSD with a sensitivity of 78% and specificity of 97% [17]. Cronbach’s alpha for the 7-item short screening scale for DSM-IV PTSD was 0.77 in this study, ranging from 0.65 in Egypt to 0.84 in Pakistan. Past week depressive symptoms (scores of 15 or more) were assessed with the “Centers for Epidemiologic Studies Depression Scale (CES-D-10)” [18]. The CES-D-10 has 10 questions, e.g., “I was bothered by things that usually don’t bother me.” Response options ranged from 0 = “Rarely (< 1 day)” to 3 = “Most (5–7 days)”, total scores ranged from 0 to 30, with higher scores presenting higher depressive symptom scores [18]. Cronbach’s alpha for the CES-D-10 was 0.74 in this sample, ranging from 0.62 in Bangladesh to 0.80 in India. The CES-D-10 has been shown to be able to correctly identify clinical depression in adolescent and adult samples [18], and the psychometric properties of the CES-D-10 have been described in this study population in university students in low- and middle-income countries [19].

### Exposure variables

#### Serious physical injury

At first, university students were shown the definition of a serious injury as follows: “An injury is serious when it makes you miss at least one full day of usual activities (such as college, sports, or a job) or requires treatment by a doctor or nurse.” [20].

In the following four questions are asked about serious injury:

1. “During the past 12 months, what were you doing when the most serious injury happened to you?” (Response options: “I was not seriously injured during the past 12 months; playing or training for a sport; walking or running, but not as part of playing or training for a sport; riding a bicycle, scooter; riding a motor cycle; riding or driving in a car or other motor vehicle; doing any paid or unpaid work, including housework, yard work, or cooking; Nothing; Something else.”) [20].
2. “During the past 12 months, what was the major cause of the most serious injury that happened to you?” (Response options: “I was not seriously injured during the past 12 months; I was in a motor vehicle accident or hit by a motor vehicle; I was on a motor cycle; I fell; something fell on me or hit me; I was fighting with someone; I was attacked, assaulted, or abused by someone; I was in a fire or too near a flame or something hot; Animal bite; Something else caused my injury.”) [20].
3. “During the past 12 months, how did the most serious injury happen to you?” (Response options: “I was not seriously injured during the past 12 months; I hurt myself by accident; someone or something else hurt me by accident; I hurt myself on purpose; Someone else hurt me on purpose.”) [20].
4. “During the past 12 months, what was the most serious injury that happened to you?” (Response options: “I was not seriously injured during the past 12 months; I had a broken bone or a dislocated joint; I had a cut, puncture, or stab wound; I had a concussion or other head or neck injury, was knocked out, or could not breathe; I had a gunshot wound; I had a bad burn; I lost all or part of a foot, leg, hand, or arm; Something else happened to me.”) [20].

### Control variables

Based on a literature review [3, 11–13, 21], control variables included age group, sex, wealth status, country income, social support, and heavy alcohol use. Wealth status was measured based on rating one’s family background as “wealthy (within the highest 25% in your country in terms of wealth), quite well off (within the 50–75% range for your country), not very well off (within the 25–50% range for your country), or quite poor (within the lowest 25% in your country in terms of wealth)” [22]. We subsequently classified low wealth status as not very well off or quite poor and high wealth status as wealthy or quite well off.

Country income was classified according to the World Bank list of economies in 2013 into low-income, lower middle-income, upper middle-income, and high-income countries [23]. Countries participating in this study were further subdivided into low- or lower middle-income countries and upper middle-income or high-income countries.

*Social support* was sourced from three items of the “Social Support Questionnaire” (SSQ) [24]. For example, “I feel that there is no one, I can share my most private concerns.” (responses ranged from 1 = completely true to 4 completely false). Scores of the 3 items were summed resulting into 3–12 scores, with higher scores representing higher social support. Total scores were dichotomised into 3–8 scores indicating low and 9–12 scores showing high social support. Cronbach’s alpha for the 3-item SSS index was 0.94 in this sample. *Past-month heavy alcohol use:* “How often do you have (for men) five or more and (for women) four or more drinks on one occasion?” [25]. Response options ranged from 1 = daily or almost daily to 5 = never. Past-month heavy drinking was defined as daily or almost daily, weekly or monthly having (for men) five or more and (for women) four or more drinks on one occasion.

### Data analysis

Descriptive calculations included frequency, median, and interquartile percentiles. Pearson Chi-square statistics were used to test for differences in proportions. Logistic regression, adjusted for age group, sex, wealth status, country, social support, and heavy alcohol use, was used to estimate the associations between injury prevalence, circumstances, causes and type and PTSD and depressive symptoms. Giving the clustered nature of the data, “country was entered as the primary sampling unit for survey analysis in STATA (StatCorp LP, College Station, TX) in order to achieve accurate confidence intervals.” Explanatory variables were free from multicollinearity as measured by the variance inflation factor (VIF < 1.8). Model assumptions were checked with residual plots, and the overall fitness of the models was checked with the Hosmer–Lemeshow goodness-of-fit test. The proportion of missing data was under 2.3% for all variables used in the analysis. Results from the logistic regression analyses are reported as odds ratios (ORs) with 95% confidence intervals (CIs). A significance level of *p* < 0.05 was considered significant, missing values were excluded from the analysis.

## Results

### Descriptive statistics

The sample consisted of 18,382 university students (20 years median age, IQR = 19–22), 58.7% were females, 53.6% rated their wealth status as high, 65.7% received high social support, 11.9% were heavy alcohol users and 53.5% lived in low- or lower middle-income countries. The overall prevalence of past 12-month injuries was 24.7%, which was higher among male students, those residing in upper middle- or high-income countries, those who had low social support, and those who engaged in heavy alcohol use. The prevalence of PTSD symptoms was 21.3% and depressive symptoms 13.3% (see Table 1).

**Table 1:** Characteristics of the sample by injury prevalence (N = 18,382)

Variable (# missing cases)	Total sample	Injury = yes	Injury = no	χ2 value	*p* value	Cramer’s V
	N (%)	%	%			
Age group (in years) (#0)						
18–19	6232 (33.8)	33.3	34.1	5.71	0.058	0.02
20–21	6861 (37.4)	38.8	36.8			
22–30	5289 (28.8)	27.9	29.1			
Sex (#53)						
Female	10,759 (58.7)	50.4	61.4	169.76	< 0.001	0.10
Male	7570 (41.3)	49.6	38.6			
Wealth status (#56)						
Low	8491 (46.4)	47.2	46.0	2.00	0.157	0.01
High	9835 (53.6)	52.8	54.0			
Country income (#0)						
Low/lower middle	9842 (53.5)	47.9	55.4	77.02	< 0.001	0.07
Upper middle/high	8540 (46.5)	52.1	44.6			
Social support (#420)						
Low	6168 (34.3)	38.9	32.9	53.80	< 0.001	0.06
High	11,794 (65.7)	61.1	67.1			
Heavy alcohol use (#0)						
No	16,194 (88.1)	84.6	89.3	71.91	< 0.001	0.06
Yes	2188 (11.9)	15.4	10.7			
PTSD symptoms (#283)						
No	14,244 (78.7)	74.3	80.2	66.10	< 0.001	0.06
Yes	3855 (21.3)	25.7	19.8			
Depressive symptoms (#0)						
No	15,943 (86.7)	83.4	87.8	58.41	< 0.001	0.06
Yes	2439 (13.3)	16.6	12.2			

### Associations between physical injury and PTSD and depressive symptoms

In adjusted logistic regression analysis, past-12 month physical injury was positively associated with PTSD symptoms (Adjusted Odds Ratio-AOR: 1.35, Confidence Interval-CI: 1.18–1.56) and depressive symptoms (AOR: 1.49, CI 1.32–1.67). Regarding the circumstances of injury, “riding a bicycle or scooter” had the highest AOR (2.11) for PTSD symptoms and the highest AOR (2.28) for depressive symptoms, followed by “doing any paid or unpaid work” AOR (1.68) for PTSD symptoms and AOR (1.89) for depressive symptoms, and “walking or running, but not as part of playing or training for a sport” AOR (1.46) for PTSD and AOR (1.62) for depressive symptoms, and “riding or driving in car or other motor vehicle” AOR (1.75) for depressive symptoms. “Playing or training for a sport” and “riding a motor cycle” were neither significantly associated with PTSD symptoms nor depressive symptoms.

In terms of the major cause of the most serious injury, having been attacked, assaulted, or abused had the highest AOR (2.65) for PTSD symptoms and the highest AOR (2.53) for depressive symptoms. Being “in a fire or near a flame or something hot” had the second highest AOR (2.20) for PTSD symptoms and “something fell on me or hit me” had the third highest AOR (1.60) for depressive symptoms. Being involved in fighting had the third highest AOR (1.76) for PTSD symptoms and the second highest AOR (2.01) for depressive symptoms, and fall had the fourth highest AOR (1.37) for PTSD symptoms and AOR (1.37) for depressive symptoms. Being in a motor vehicle accident, riding on a motor cycle and animal bites were neither significantly associated with PTSD symptoms nor depressive symptoms (see Table 2).

**Table 2:** Associations between physical injury prevalence, circumstances, and causes of most serious physical injury in the past 12 months and PTSD and depressive symptoms (N = 18,382)

Variable (#missing cases)	Response	Sample	PTSD symptoms	Nagelkerke R square	Depressive symptoms	Nagelkerke R square
		N (%)	AOR (95% CI)^1^		AOR (95% CI)^1^	
*Annual injury prevalence*						
Most serious injury in the past 12 months (#0)	No	13,834 (75.3)	1 (Reference)	0.04	1 (Reference)	0.03
	Yes	4548 (24.7)	1.35 (1.18, 1.56)***		1.49 (1.32, 1.67)***	
*What were you doing when the most serious injury happened?*						
Playing or training for a sport (#0)	No	17,200 (93.6)	1 (Reference)	0.03	1 (Reference)	0.03
	Yes	1182 (6.4)	0.92 (0.74, 1.14)		1.09 (0.91, 1.30)	
Walking or running, but not as part of playing or training for a sport (#1)	No	17,908 (97.4)	1 (Reference)	0.03	1 (Reference)	0.03
	Yes	473 (2.6)	1.46 (1.03, 2.07)*		1.62 (1.25, 2.08)***	
Riding a bicycle, scooter (#1)	No	18,215 (99.1)	1 (Reference)	0.03	1 (Reference)	0.03
	Yes	166 (0.9)	2.11 (1.30, 3.43)**		2.28 (1.41, 3.68)**	
Riding a motor cycle (#1)	No	18,039 (98.1)	1 (Reference)	0.03	1 (Reference)	0.03
	Yes	342 (1.9)	1.10 (0.79, 1.53)		0.61 (0.26, 1.40)	
Riding or driving in a car or other motor vehicle (#1)	No	18,208 (99.1)	1 (Reference)	0.03	1 (Reference)	0.03
	Yes	173 (0.9)	1.43 (0.90, 2.28)		1.75 (1.11, 2.78)*	
Doing any paid or unpaid work, including housework, yard work, or cooking (#1)	No	17,992 (97.9)	1 (Reference)	0.03	1 (Reference)	0.03
	Yes	389 (2.1)	1.68 (1.38, 2.05)***		1.89 (1.49, 2.40)***	
Nothing (#1)	No	17,220 (93.7)	1 (Reference)	0.03	1 (Reference)	0.03
	Yes	1161 (6.3)	1.13 (0.81, 1.59)		1.11 (0.87, 1.41)	
Something else (#1)	No	17,720 (96.4)	1 (Reference)	0.03	1 (Reference)	0.03
	Yes	661 (3.6)	1.75 (1.43, 2.15)***		1.51 (1.22, 1.88)***	
*What was the major cause of the most serious injury*						
I was in a motor vehicle accident or hit by a motor vehicle (#108)	No	17,992 (98.5)	1 (Reference)	0.03	1 (Reference)	0.03
	Yes	282 (1.5)	1.39 (0.99, 1.96)		1.37 (0.75, 2.50)	
I was on a motor cycle (#108)	No	17,949 (98.2)	1 (Reference)	0.03	1 (Reference)	0.03
	Yes	325 (1.8)	1.38 (0.92, 2.07)		0.92 (0.42, 2.03)	
I fell (#108)	No	17,166 (93.9)	1 (Reference)	0.03	1 (Reference)	0.03
	Yes	1108 (6.1)	1.37 (1.15, 1.64)***		1.37 (1.14, 1.04)**	
Something fell on me or hit me (#108)	No	17,963 (98.3)	1 (Reference)	0.03	1 (Reference)	0.03
	Yes	311 (1.7)	1.35 (0.91, 2.02)		1.60 (1.16, 2.19)**	
I was fighting with someone (#108)	No	18,114 (99.1)	1 (Reference)	0.03	1 (Reference)	0.03
	Yes	160 (0.9)	1.76 (1.17, 2.66)**		2.01 (1.30, 3.12)**	
I was attacked, assaulted, or abused by someone (#108)	No	18,196 (99.6)	1 (Reference)	0.03	1 (Reference)	0.03
	Yes	78 (0.4)	2.65 (1.77, 3.98)***		2.53 (1.44, 4.47)**	
I was in a fire or too near a flame or something hot (#108)	No	18,184 (99.5)	1 (Reference)	0.03	1 (Reference)	0.03
	Yes	90 (0.5)	2.20 (1.36, 3.56)**		1.48 (0.94, 2.32)	
Animal bite (#108)	No	18,158 (99.4)	1 (Reference)	0.03	1 (Reference)	0.03
	Yes	116 (0.6)	1.05 (0.59, 1.87)		1.20 (0.53, 2.73)	
Something else caused my injury (#108)	No	17,080 (93.5)	1 (Reference)	0.03	1 (Reference)	0.03
	Yes	1194 (6.5)	1.40 (1.17, 1.69)***		1.44 (1.21, 1.72)***	

*AOR* adjusted odds ratio, *CI* confidence interval; adjusted for age, sex, wealth status, country, social support and heavy alcohol use;

*p < .05, **p < .01, ***p < .001

Regarding how the most serious injury happened, someone else hurt me on purpose had the highest AOR (2.06) for PTSD symptoms and “hurt myself on purpose” had the highest AOR (2.72) for depressive symptoms, followed by “hurt myself on purpose” (AOR = 1.99), “someone or something else hurt me by accident” (AOR = 1.31) and “hurt myself by accident (AOR = 1.29) for PTSD symptoms, and followed by someone else hurt me on purpose (AOR = 1.68), “someone or something else hurt me by accident” (AOR = 1.31) and “hurt myself by accident” (AOR = 1.23) for depressive symptoms. As for the major type of injury, gunshot wounds had the highest AOR (2.28) for PTSD symptoms as well as the highest AOR (3.31) for depressive symptoms, followed by cut, puncture or stab wound (AOR = 1.64) for PTSD symptoms, and followed by concussion or other head or neck injury (AOR = 2.28), and “broken bone or a dislocated joint” (AOR = 1.47) for depressive symptoms. Having had “a bad burn” and “lost all or part of a foot, leg, hand, or arm” were neither significantly associated with PTSD symptoms nor depressive symptoms (see Table 3).

**Table 3:** Associations between how the injury happened and type of most serious physical injury in the past 12 months and PTSD and depressive symptoms

Variable (# missing cases)	Response	Sample	PTSD symptoms	Nagelkerke R square	Depressive symptoms	Nagelkerke R square
		N (%)	AOR (95% CI)		AOR (95% CI)	
*How did the most serious injury happen to you?*						
I hurt myself by accident (#165)	No	16,469 (90.4)	1 (Reference)	0.03	1 (Reference)	0.03
	Yes	1748 (9.6)	1.29 (1.13, 1.47)***		1.23 (1.03, 1.46)*	
Someone or something else hurt me by accident (#165)	No	17,284 (94.9)	1 (Reference)	0.03	1 (Reference)	0.03
	Yes	933 (5.1)	1.31 (1.05, 1.64)*		1.31 (1.00, 1.71)*	
I hurt myself on purpose (#165)	No	18,067 (99.2)	1 (Reference)	0.03	1 (Reference)	0.03
	Yes	150 (0.8)	1.99 (1.32, 2.99)**		2.72 (1.89, 3.91)***	
Someone else hurt me on purpose (#165)	No	17,906 (98.3)	1 (Reference)	0.03	1 (Reference)	0.03
	Yes	311 (1.7)	2.06 (1.42, 2.99)***		1.68 (1.36, 2.08)***	
*What was the most serious injury that happened to you?*						
I had a broken bone or a dislocated joint (#0)	No	17,617 (95.8)	1 (Reference)	0.03	1 (Reference)	0.03
	Yes	765 (4.2)	1.27 (0.94, 1.71)		1.47 (1.19, 1.81)***	
I had a cut, puncture, or stab wound (#0)	No	17,696 (93.3)	1 (Reference)	0.03	1 (Reference)	0.03
	Yes	686 (3.7)	1.64 (1.27, 2.13)***		1.40 (1.08, 1.82)*	
I had a concussion or other head or neck injury, was knocked out, or could not breathe (#0)	No	18,192 (99.0)	1 (Reference)	0.03	1 (Reference)	0.03
	Yes	190 (1.0)	1.61 (0.95, 2.74)		2.28 (1.55, 3.35)***	
I had a gunshot wound (#0)	No	18,345 (99.8)	1 (Reference)	0.03	1 (Reference)	0.03
	Yes	37 (0.2)	2.28 (1.05, 4.97)*		3.31 (1.29, 8.49)*	
I had a bad burn (#0)	No	18,225 (99.1)	1 (Reference)	0.03	1 (Reference)	0.03
	Yes	157 (0.9)	1.30 (0.92, 1.83)		1.16 (0.73, 1.83)	
I lost all or part of a foot, leg, hand, or arm (#0)	No	18,284 (99.5)	1 (Reference)	0.03	1 (Reference)	0.03
	Yes	98 (0.5)	1.12 (0.85, 1.47)		0.34 (0.09, 1.28)	
Something else happened to me (#0)	No	17,107 (93.1)	1 (Reference)	0.03	1 (Reference)	0.03
	Yes	1275 (6.9)	1.40 (1.17, 1.67)***		1.35 (1.10, 1.65)***	

*AOR* adjusted odds ratio, *CI* confidence interval; adjusted for age, sex, wealth status, country, social support and heavy alcohol use

*p < .05, **p < .01, ***p < .001

## Discussion

Based on our knowledge, this multicountry study is the first to assess the association between having had a serious physical injury and mental health outcomes (PTSD and depression symptoms) in a large sample of university students in Africa, Asia and the Americas, and demonstrates a relevant research contribution on the mental health outcomes of physical injury. In this study of university students across 26 countries, compared to noninjured students, injured students had a significantly higher prevalence of PTSD and depressive symptoms. These findings are largely consistent with previous clinical research and population studies among adolescents, adults, and older adults [6, 9–13] that have linked physical injury and poor mental health in terms of depressive and PTSD symptoms, confirming our hypothesis.

Various factors may be responsible for the physical injury-poor mental health association among university students, predominantly from low- and middle-income countries [8]. A serious physical injury may be perceived as a traumatic event leading to PTSD symptoms [6], or associated with pain and/or disability leading to depressive symptoms [11–13]. Another contributing factor could be the lack of availability of both care for injury and mental health in the low-resourced study countries [13]. Furthermore, the relationship between physical injury and poor mental health may also be bidirectional [8, 13, 26], such that PTSD and/or depressive symptoms act as risk factors for injury.

Differences in the different types, causes and circumstances of physical injuries in relation to PTSD and depressive symptoms were observed. For example, having had a gunshot wound, having been “attacked, assaulted, or abused by someone”, and riding a bicycle or scooter resulting in injury had the highest odds ratios for PTSD and depressive symptoms. Similar results in terms of gunshot wounds and having been attacked, assaulted, or abused by someone were found in a multicountry study among adolescents [13]. Unlike the multicountry study among adolescents [13], this study did not find a significant association between physical injuries from playing or training for sport and motor vehicle or motorcycle accidents and PTSD and depressive symptoms. This result may be surprising, especially since in a review of child and adolescent victims of road traffic accidents a high prevalence of PTSD (19.95%) was found [27]. However, in a cross-sectional survey among adolescents aged 15 years and older, victims of motor vehicle crashes also did not have a significantly higher prevalence of depression nor PTSD than nonvictims of motor vehicle crashes [28]. It is possible that the development of PTSD and depressive symptoms depends on a history of traumatic events beyond the motor vehicle crash [28, 29], the extent of the physical injury, “as well as victim reports of their perceptions of how much danger they perceived at the time of the accident and the degree of life threat (fear of dying) they perceived.” [30]. Furthermore, Reiland and Clark [31] tried to show in a study among university students that the interpersonal rather than the non-interpersonal nature of the event type had higher event centrality and greater impact on PTSD and depressive symptoms. Interpersonal injury event types in this study may include being involved in physical fighting, being “attacked, assaulted, or abused by someone”, hurting oneself on purpose or someone else was hurt on purpose, which were all significantly associated with PTSD and depressive symptoms, while non-interpersonal event types in this study may include physical injury from an accident related to motor vehicle or motorcycle crash, animal bite, and burns, which were all not significantly associated with PTSD and depressive symptoms. Following this reasoning, it is possible that students in selecting the most serious injury in the past 12 months chose to report on the interpersonal over the non-interpersonal injury event type.

Possible implications of the study findings are that students who have experienced serious physical injury could be screened for PTSD and depressive symptoms following the injury occurrence [12]. University campus health workers could be made aware that students who were injured as a result of a gunshot wound, having been “attacked, assaulted, or abused by someone”, and riding a bicycle or scooter are especially at risk for PTSD and/or depressive symptoms. Furthermore, the prevention of physical injury among university students may assist in reducing PTSD and depressive symptoms. The most prevalent types of injuries found in this study among university students were related to “playing or training for sport” or “walking or running not as part of a sport”, while falls, fractures, or dislocated joints and cuts, puncture or stab wounds were also common. Relevant strategies to prevent sport and other injuries may involve health education and advocacy in the prevention of injuries among young people [3].

### Study limitations and strength

Study limitations included the cross-sectional survey design, the limitation to university students, and purposely selected universities. Therefore, we were not able to determine the direction of the relationship between physical injury and mental health outcomes (PTSD and depressive symptoms) and generalize the findings. Data were collected by self-report which may have biased responses. In addition, certain aspects of physical injury, such as the frequency, the perceived seriousness, extent of disability, location and care seeking behaviour, were not assessed, which should be included in future investigations. Furthermore, PTSD and depressive symptoms were only assessed with screening tools and not a clinical psychiatric interview. The strength of the study was that uniform assessment measures were administered across university students in 26 countries.

## Conclusion

Results of a large multicountry study indicate that compared to students who had not sustained a serious physical injury in the past 12 months, students with an injury had significantly higher PTSD and depressive symptoms. Mental health support of students who sustained physical injuries may prevent PTSD and depressive symptoms. University campus health care staff should be made aware of the comorbidity of physical injury with PTSD and depressive symptoms. Country injury prevention policies should be reinforced to reduce both injuries and mental ill health.

## Data Availability

The data for the current study will not be shared publicly as participants were informed at the time of providing consent that only researchers involved in the project would have access to the information they provided.

## Abbreviations

CES-D-10: 10 Item Center for Epidemiologic Studies Depression Scale
IQR: Interquartile range
PTSD: Posttraumatic stress disorder
